# Diagnostic biomarkers in tear fluid: from sampling to preanalytical processing

**DOI:** 10.1038/s41598-021-89514-8

**Published:** 2021-05-12

**Authors:** Franziska Bachhuber, André Huss, Makbule Senel, Hayrettin Tumani

**Affiliations:** 1 Department of Neurology, University Hospital Ulm, Ulm, Germany; 2 Specialty Hospital of Neurology Dietenbronn, Schwendi, Germany

**Keywords:** Diagnostic markers, Visual system, Diagnostic markers

## Abstract

Tear fluid is receiving growing attention as a source for novel diagnostic biomarkers. Multiple techniques are available for its collection and impact the composition of acquired samples. We sought to provide a direct comparison of two collection methods with regard to implementation, acceptance, and impact on sample composition. Tear fluid was collected from fifteen healthy volunteers with capillary tubes and Schirmer strips and analyzed for total protein and IgG concentrations. Sampling parameters and perception by test persons were compared. The use of capillary tubes was more convenient for the participants while causing more effort for the collector. Tear flow rates as well as the relative and absolute amount of IgG were higher when Schirmer strips were used. Consecutive collections with Schirmer strips significantly influenced tear flow rates, IgG, and protein concentrations. A moderate correlation was observed between tear flow rates and IgG concentrations for both methods. Samples collected with both methods can be analyzed by isoelectric focusing, a potential diagnostic application in the field of neurology. The specific advantages and limitations of tear fluid sampling with either capillary tubes or Schirmer strips demonstrate the need for a thorough investigation of collection methods with regard to the application of interest.

## Introduction

Biomarkers are essential for the prediction and diagnosis of diseases as well as for the monitoring of progression and therapy. Besides well-established markers that set the gold standard in clinical routine there is an ongoing search for new approaches to enhance and extend the diagnostic spectrum. Easily accessible biological fluids provide promising opportunities for further research. Tear fluid offers non-invasive sample acquisition and high protein content and therefore garners continuously growing interest for the discovery of novel biomarkers^1,2^. So far, the analysis of tear fluid has opened interesting possibilities for research and diagnostics in many fields beyond ophthalmology, including neurological diseases such as multiple sclerosis^3–10^, Parkinson’s disease^11–13^, and Alzheimer dementia^14,15^.

The most commonly described techniques for the collection of tear fluid are Schirmer strips and capillary tubes^16^. For these methods, however, numerous protocols have been applied, potentially influencing the composition and quality of obtained samples and introducing a considerable variability among studies^17–19^. Accordingly, contradicting results in tear fluid research have been attributed to differences in sample collection and handling procedures^20^. Besides the aspect of sample quality, the applicability of a tear fluid test in clinical practice depends on its feasibility and acceptance by patients. The optimal collection method should be fast and simple for the investigator as well as convenient and painless to the patient.

The concentration of proteins in tear fluid can be influenced by various factors including reflex tearing, collection method, and post-collection sample processing. Based on the rate of their secretion, tears can be categorized into basal and reflex tears. Basal tears constitute the lacrimal film on the surface of the cornea and are continuously produced at a rate of 0.5–2.2 µl/min^21,22^. Reflex tearing, caused by mechanical, sensory or emotional stimulation, has been shown to significantly reduce the concentration of many tear fluid proteins^23^. Moreover, the use of Schirmer strips has been reported to influence sample composition due to mechanical contact with cornea and conjunctiva^24,25^. Additional variability is introduced during the recovery of tears from the collection tool, a process in which different proteins are lost to varying extent^26,27^.

Considering the various parameters influencing the molecular composition of tear fluid samples, it is essential to investigate different collection techniques with regard to their impact on the quality and composition of acquired samples, especially prior to quantitative analyses. In our study, we were interested in total protein and immunoglobulin G (IgG) concentrations. The tear film is part of the mucosal immune system, contains immunoglobulins, and provides further possibilities for research questions involving humoral immunity^28–30^.

In the field of neurology, it is an important part of the diagnostic workup to detect signs of inflammation and infection within the central nervous system (CNS). A gold standard method for the detection of immunoglobulin synthesis within the CNS is the investigation of oligoclonal IgG bands (OCB) in the cerebrospinal fluid (CSF) of patients. It has been proposed that OCB can not only be detected in CSF, but also in tear fluid^5–9^. The analysis of tear fluid for OCB would present an interesting non-invasive diagnostic approach that demands further investigation.

This exploratory study aimed to revise the protocols of tear fluid collection by capillary tubes and Schirmer strips. The goal was to assess these methods and to compare them with a focus on two major aspects, the convenience and applicability of the collection techniques as well as their influence on tear fluid composition, particularly on two parameters, total protein and IgG concentration.

## Results

### Procedures for the collection of tear fluid samples

**Figure 1:**
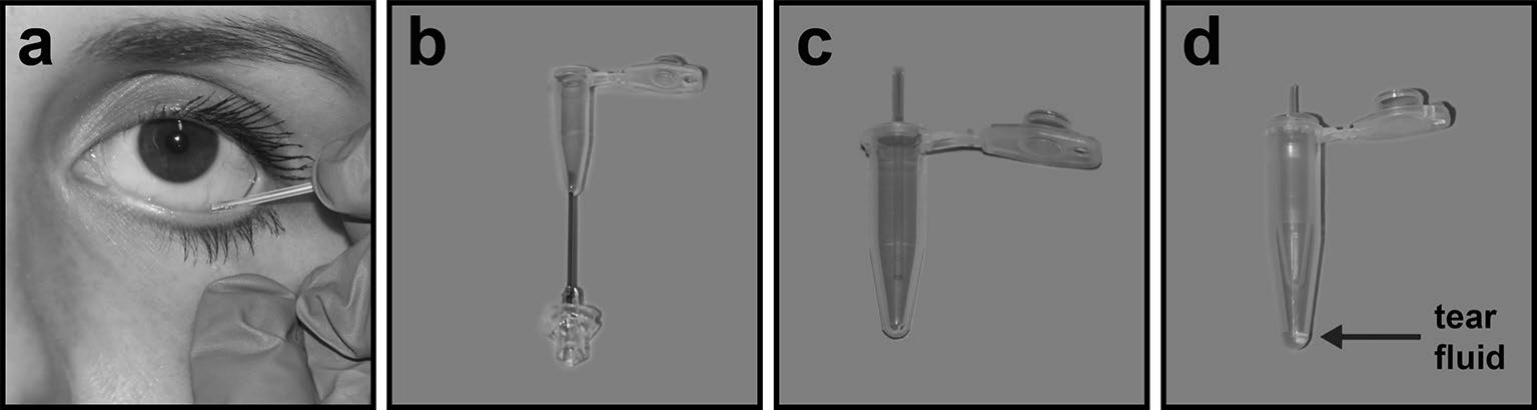
Tear fluid collection with plastic capillary tubes. (**a**) An end-to-end plastic capillary tube with a capacity of 10 μl is placed at the lower conjunctival fornix until the desired volume of tear fluid is collected utilizing capillary action. (**c**) The capillary is transferred to a punctured 0.5 ml tube (**b**) and (**d**) centrifuged into a 1.5 ml tube in order to retrieve the tear fluid.

**Figure 2:**
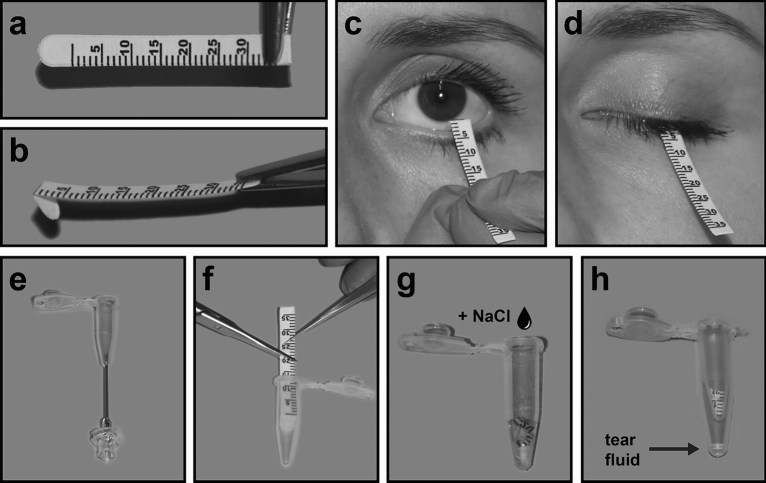
Tear fluid collection with Schirmer strips. (**a**) Schirmer strips are small pieces of filter paper with an imprinted graduated scale. (**b**) Strips are bent on the zero line at an angle of 120 degrees and (**c**) placed beneath the eyelid at a median position. (**d**) Eyes are closed for 5 min. (**f**) The soaked part of the Schirmer strip is cut and placed in a punctured 0.5 ml tube (**e**). (**g**) A known volume of 0.9% NaCl, proportional to the collected volume, is added for an incubation time of 2 h. (**h**) The tube containing the Schirmer strip is centrifuged into a 1.5 ml tube in order to retain the fluid.

This investigation focused on the two most commonly used methods for tear fluid collection: capillary tubes and Schirmer strips^16^. The main focus was the collection of tear fluid without stimulation at a volume allowing for further laboratory analysis in a way that can be implemented in clinical routine and is convenient to the patient. Sampling with capillary tubes aimed to collect a volume of 10 µl in a timeframe of up to ten minutes. The tear fluid samples were extracted from the capillaries by centrifugation (Fig. 1). Plastic rather than glass capillaries were used to minimize the risk of injury as well as concerns of probands. In the second technique, tear fluid was absorbed by Schirmer strips^31^ (Fig. 2). Due to sparse recovery of tear volume after centrifugation, a diffusion based elution step was included in the protocol in accordance with the findings of Denisin et al.^26^.

Tear fluid was collected from 15 healthy volunteers using capillary tubes and Schirmer strips. Four samples were collected from each individual on the same day, first from both eyes with capillary tubes and then from both eyes with Schirmer strips with pauses between each collection. Time requirement and yield were recorded for every sample. Furthermore, the experience of pain, discomfort and foreign body sensation (FBS) was assessed (Table 1).

**Table 1:** Tear fluid collection from healthy subjects.

	Capillary tubes	Schirmer strips
Duration	6.0 (4.3–9.2) min	5.0 (3.8–5.0) min
Collected volume	10.0 (7.6–10.0) µl	10.1 (5.5–13.9) µl
Distance moistened		10.8 (5.3–15.0) mm
Flow rate	1.4 (1.0–2.3) µl/min	2.3 (1.2–3.3) µl/min
Flow rate > 2.2 µl/min	9 (30%)	15 (50%)
Foreign body sensation (FBS)	8 (27%)	13 (43%)
Duration of FBS	84 (32–120) s	180 (60–194) s
Pain	0.0 (0.0–0.2)	0.0 (0.0–2.0)
Discomfort	0.8 (0.0–2.4)	1.0 (1.0–2.9)

Capillary tube and Schirmer strip samples were collected from both eyes. Values are displayed as median and interquartile range (IQR) or quantity (percentage of total number). *n* = 30 from 15 individuals.

Both methods were safe and tolerated well with no reports of adverse effects. Tear flow rates were higher when Schirmer strips were used. The rate of basal tear flow described in the literature as 0.5–2.2 µl/min^21, 22^ was exceeded in 30% of capillary tube and 50% of Schirmer strip collections. The participants reported FBS after 21 of 60 samplings. The use of capillary tubes caused less frequent and shorter experiences of FBS. The scores for inconvenience in the form of pain or discomfort reported by the probands were higher for Schirmer strips, while the absolute values were low for both methods (Table 1).

### IgG and total protein concentrations in tear fluid samples

Samples from ten healthy volunteers were further analyzed regarding total protein and IgG concentration. For quantitative analyses in tear fluid, it is relevant to know whether similar concentrations are observed in samples collected from the left or right eye or with different methods. We observed a great heterogeneity of tear flow rates and IgG concentrations, and to a lesser extent of total protein content, for samples collected from both eyes of the same individual. The detailed values for tear flow rate, IgG and protein concentrations are listed in Supplementary Table S1. Median IgG concentrations were 27.6 (IQR 6.6–76.1) µg/ml in capillary tube samples and 59.8 (IQR 33.1–88.8) µg/ml in Schirmer strip samples. The observed protein concentration had a median of 15.0 (IQR 13.5–16.6) mg/ml for capillary tubes and 9.6 (IQR 6.8–13.8) mg/ml for Schirmer strips. The proportion of IgG concentration in relation to the total protein content was higher when Schirmer strips were used and exceeded that of capillary tubes in median by factor 4 (IQR 1.6–9.0).

### Sequential sample collection influences flow rate and sample composition when Schirmer strips are used

As a measure to prevent reciprocal influence, the sampling procedure included time to equilibrate between consecutive sample collections, in median 19.9 (IQR 17.0–30.6) min between capillary tubes, 25.8 (IQR 19.5–32.4) min between capillaries and Schirmer strips, and 21.0 (IQR 15.7–39.1) min between Schirmer strips. To investigate whether the collection sequence nonetheless contributes to the observed eye-to-eye variability, results from both eyes were compared (Table 2). In a comparison of data from the eye sampled as first (side I) and as second (side II), no significant differences were observed when samples were gathered with capillary tubes. For samples collected with Schirmer strips the tear flow rate was significantly lower for the collection on side II (*p* = 0.008) while protein and IgG concentrations were significantly higher (*p* = 0.01 and *p* = 0.02).

**Table 2:** Flow rate, total protein and IgG concentration.

	Capillary tubes			Schirmer strips		
	Tear flow (µl/min)	IgG conc. (µg/ml)	Protein conc. (mg/ml)	Tear flow (µl/min)	IgG conc. (µg/ml)	Protein conc. (mg/ml)
Side I	1.5 (1.1–3.6)	31.6 (6.9–114)	15.6 (14.2–17.7)	2.9 (1.2–4.4)	45.3 (24.7–78.8)	8.5 (6.7–12.2)
Side II	1.4 (0.9–2.3)	24.7 (2.4–73.9)	14.9 (12.2–16.2)	1.7 (1.1–3.1)	79.4 (35.0–109)	10.3 (6.6–15.2)
*p* value	0.25	0.43	0.26	0.008	0.01	0.02

Tear fluid samples were collected with capillary tubes and Schirmer strips first from one eye (side I) followed by the other eye (side II). Results were compared to investigate a potential influence of the collection sequence. Values are displayed as median and interquartile range (IQR). *n* = 15 for tear flow, *n* = 10 for IgG and protein concentration. *p* values were calculated using the two-tailed Wilcoxon matched-pairs signed rank test, significant for *p* < 0.05.

### Correlation analysis of tear flow rate, IgG, and total protein concentrations

**Figure 3:**
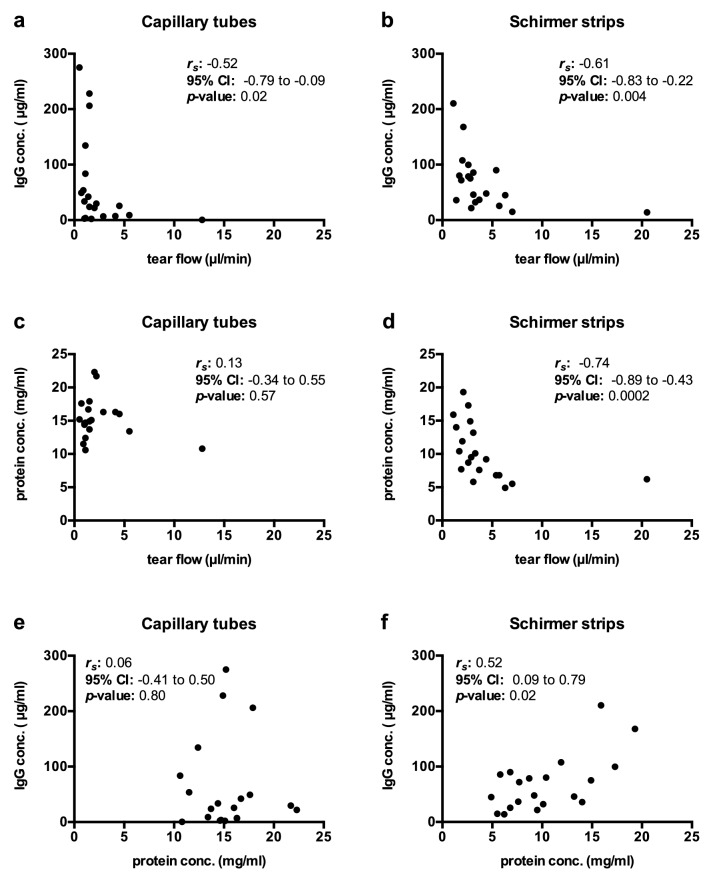
Correlation analysis for tear flow rate, IgG, and protein concentration. Spearman rank correlation was performed to investigate a potential correlation between tear flow rate, IgG concentration, and protein concentration for samples collected with capillary tubes and Schirmer strips. A moderate correlation was observed between tear flow rate and IgG concentration for capillary tubes (**a**) and Schirmer strips (**b**). A strong correlation was observed between tear flow rate and protein concentration for Schirmer strips (**d**) while no correlation was observed for capillary tubes (**c**). Between IgG and protein concentration no association was observed for capillary tubes (**e**) and a moderate association for Schirmer strips (**f**). *n* = 20, significant for *p* < 0.05. *r*_*S*_: Spearman’s rank coefficient; CI: confidence interval; conc.: concentration.

The association of tear flow rate, protein, and IgG concentration was investigated by correlation analysis. For samples collected with capillary tubes, tear flow rates showed a moderate correlation (*r*_*s*_ =  − 0.52, *p* = 0.02) with IgG concentration (Fig. 3a) and no correlation (*r*_*s*_ = 0.13, *p* = 0.57) with protein concentration (Fig. 3c). Tear flow rates of samples collected with Schirmer strips showed a moderate correlation (*r*_*s*_ =  − 0.61, *p* = 0.004) with IgG concentration (Fig. 3b) and a strong correlation (*r*_*s*_ = 0.74, *p* = 0.0002) with protein concentration (Fig. 3d). No correlation between total protein and IgG concentrations was observed for capillary tubes (*r*_*s*_ = 0.06, *p* = 0.80) (Fig. 3e) and a moderate correlation (*r*_*s*_ = 0.52, *p* = 0.02) for Schirmer strips (Fig. 3f).

### Tear fluid samples collected with both methods can be analyzed by isoelectric focusing

**Figure 4:**
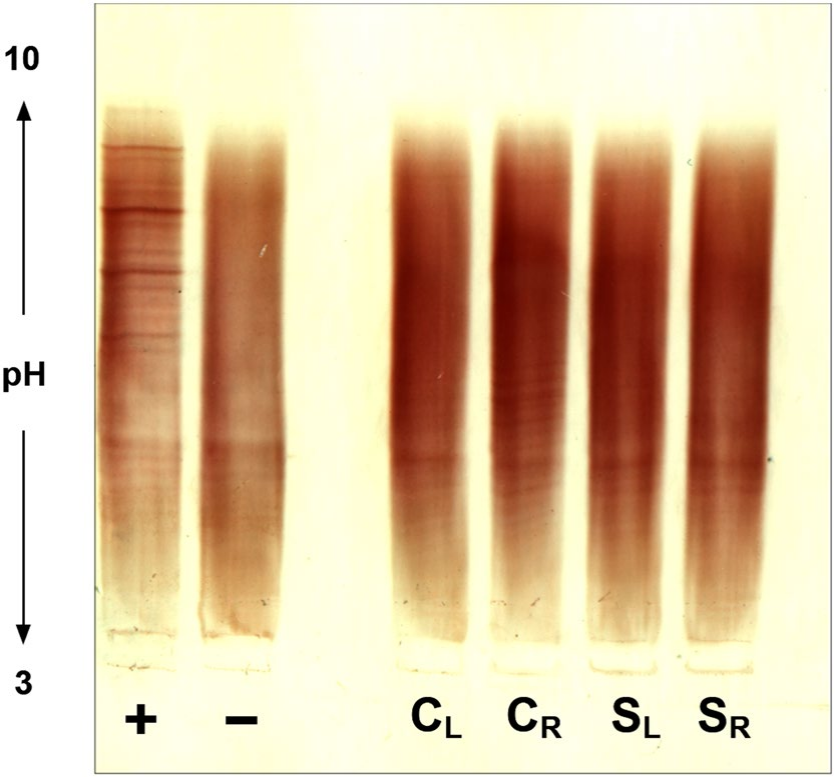
Analysis of tear fluid by isoelectric focusing for the detection of oligoclonal IgG bands. Tear fluid collected with capillary tubes (**C**) and Schirmer strips (**S**) from the left (**L**) and right (**R**) eye of a healthy subject was analyzed by isoelectric focusing and IgG was visualized in an immunoblot. Samples as well as CSF positive (**+**) and negative (−) controls were analyzed at an IgG concentration of 4 µg/ml. Distinct oligoclonal IgG bands can be found in the positive control (**+**). A clear polyclonal IgG signal was obtained in all tear fluid samples, which were negative for oligoclonal IgG bands. The section displayed was vertically cropped from both sides. The full-length blot is presented in Supplementary Figure S1.

A potential application of tear fluid in neurological diagnostics is its analysis by isoelectric focusing for the detection of OCB^5,7–9^. Tear fluid samples were subjected to IEF to investigate, whether both collection methods can be applied in the context of this specific analysis. To account for the limited sample volume, the procedure of OCB detection was adjusted to lower IgG concentrations as customary in routine CSF diagnostics. Both collection methods provided sample material that was analyzable at an IgG concentration of 4 µg/ml, which allowed a clear detection of bands in the positive control sample (Fig. 4).

## Discussion

The implementation of tear fluid testing for diagnostic biomarkers requires collection methods that are reliable, quick, accepted by patients, and robust with regard to sample quality. Protocols for the use of capillary tubes and Schirmer strips were investigated in a small cohort of healthy volunteers aiming to collect unstimulated tear fluid with a volume sufficient for further analysis of tear fluid proteins. In addition to the investigation of protein levels, we provide descriptive data on sample collection as well as patient-reported values on the perception of the collection methods.

Both methods were safe and well tolerated. In an intra-individual comparison, the capillary tube method was considered more comfortable and caused less frequent and shorter foreign body sensations. Besides the more convenient sample collection for the patient, an advantage of capillary tubes is their simple post-collection handling. The complete volume of undiluted tear fluid can be collected in a quick centrifugation step. In contrast, Schirmer strips require tear fluid to be extracted. The strips retain proteins to varying extent, depending on their molecular properties^24,26^. Published extraction protocols include centrifugation^32^, centrifugation combined with a washing step^9,16^, or diffusion-based elution^7,33^ where the choice of solvent, time, temperature, and volume influences extraction efficiency^27^. All extraction strategies experience sample loss and affect sample composition^24,27^.

An advantage of Schirmer strips, however, is their simple application. While the use of capillary tubes requires experience and is time consuming, Schirmer strips can be easily implemented in clinical routine, require less handling during sample collection and additionally provide information about the dry-eye status of the patient. In a comparative study, Posa et al.^32^ described the easy applicability of Schirmer strips as a decisive criterion in favor of their use.

An important question of this exploratory study was the investigation of consistency between samples collected from different eyes of the same individual or with the different methods. High intra-individual variability was observed with regard to both aspects. Overall, total protein concentrations were higher in samples collected with capillary tubes. In contrast, IgG concentrations were higher in samples collected with Schirmer strips, regarding absolute levels as well as relative amount in relation to the total protein concentration.

Interestingly, we observed significant differences in sequential sample collections with Schirmer strips but not with capillary tubes. In a comparison of tear fluid collected from both eyes with the same method, lower tear flow rates were observed on side II for Schirmer strips. In line with the observed trend to an inverse relationship with tear flow rate, the median IgG and protein levels were significantly higher in these samples. This finding reveals difficulties with consecutive sample collections or pooling of samples when Schirmer strips are used. Furthermore, samples collected with Schirmer strips showed moderate or strong correlations between tear flow rate, IgG and protein concentrations while such an association was only observed between tear flow rates and IgG concentrations for capillary tubes.

Differences between the two methods might be related to the distinct modes of sample acquisition. As discussed, higher tear flow rates in Schirmer strips or protein loss during extraction might influence protein levels. Furthermore, the direct contact of Schirmer strips with the cornea and conjunctiva involves mechanical irritation and has been described to induce vascular transudation or cell damage, and thus leakage of cellular^24^ or plasma^25^ proteins into the collected sample. In contrast, contact with any surface of the eye is avoided when using capillary tubes.

An inverse relationship between tear flow rate and the levels of specific proteins has been described in the literature—especially a heavily stimulated tear flow over 50 µl/min leads to a significant reduction in the levels of many proteins^23^. In order to avoid dilution and distortion of samples, we aimed to collect non-stimulated tears at flow rates that do not substantially deviate from the estimated basal tear flow of 0.5–2.2 µl/min^21,22^. However, despite careful effort to prevent reflex tearing, the described range of basal tear flow was exceeded in 30% of capillary tube and in 50% of Schirmer strip collections. Previous studies mainly focused on the comparison of basal tears to heavily stimulated reflex tears. We were interested in the question, whether there is a relationship between IgG concentration and tear flow rate in tears that were collected without intentional stimulation. The observed moderate negative correlation between IgG concentrations and tear flow rates for samples collected with both methods indicates that IgG levels in unstimulated tears are influenced by, but not solely dependent on, tear flow rates.

A strong association between the concentration of specific proteins and parameters such as tear flow rate or total protein content would allow their use as a reference value to account for variations in the collection process. However, we did not observe a strong correlation between IgG concentrations and tear flow or protein levels, precluding a prediction of IgG concentrations on their basis. The variability of tear flow and the association between flow rates and protein concentrations nevertheless raise the question for the necessity of a reference parameter to correct for variability introduced by tear flow rates. In our study, we were not able to identify a suitable parameter as reference basis. For further investigations the site of production of the investigated protein could be of interest. A protein produced in the lachrymal apparatus might require a tear specific protein as reference parameter while a blood-borne protein might benefit from a blood specific reference parameter.

A potential application of tear fluid analysis in neurological research and diagnostics is the investigation of OCB. Previous studies on OCB in tear fluid have employed a variety of collection and handling methods and came to differing conclusions on their applicability for OCB analysis as well as on the occurrence of OCB in tear fluid^5–9,34–36^. Here, we demonstrated the suitability of samples collected with the two most relevant methods for an analysis by isoelectric focusing, thereby allowing a deeper investigation of tear fluid for OCB in subsequent studies.

Both suggested protocols allowed the collection and analysis of small volumes of tear fluid. The samples can be acquired quickly, easily, repeatedly and without the need for special equipment or invasive procedures. As such, tear fluid analysis opens new possibilities for research and diagnostics in many fields beyond ophthalmology. Importantly, different sampling methods have their own specific advantages and limitations and influence the yield of the analyte of interest. Hence it is beneficial to compare different methods of tear fluid collection with regard to their suitability for the specific research question.

## Methods

### Ethical approval

The research related to human use has complied with all relevant national regulations, institutional policies, and was conducted in accordance with the tenets of the Declaration of Helsinki. The study protocol was approved by the Ethics Committee of Ulm University (application number 280/16). All subjects gave written informed consent.

### Human subjects

Tear fluid was collected from 15 healthy volunteers (7 female, 8 male). The median age was 30.4 years with an interquartile range from 29.4 to 45.1 years and a range from 26.8 to 58.3 years. Exclusion criteria were known or apparent inflammatory diseases of the eye and contact lens use.

### Tear fluid collection

Tear fluid samples were collected with uncoated 10 µl plastic capillaries (Kabe Labortechnik GmbH, Nümbrecht-Elsenroth, Germany) or with Schirmer strips (Optitech Eyecare, Allahabad, India) from the lower conjunctival fornix without stimulation or anesthesia. Tear fluid was extracted from capillaries by centrifugation (1 min, 13,000 rpm). Schirmer test I was performed in accordance with the Tear Film and Ocular Surface Society Dry Eye Workshop guidelines on diagnostic methodologies^31^. Strips were bent as indicated, inserted at a median position and used with closed eyes for five minutes. To elute tear fluid from Schirmer strips, these were incubated with 0.9% sodium chloride (NaCl; Braun, Melsungen, Germany; 3570160) at room temperature for two hours at 300 rpm (ThermoMixer comfort, Eppendorf, Hamburg, Germany) and subsequently collected by centrifugation. Until further use, samples or strips were stored at − 80 °C. The perception of pain and discomfort was assessed on a questionnaire using a numeric rating scale (minimum 0, maximum 10) together with the experience and duration of foreign body sensations after collection. Time, duration, and tear volume were recorded by the collector.

### Total protein concentration

The total protein concentration was assessed by Bradford assay (Thermo Fisher Scientific, Waltham, USA; 23236) in a microplate format. In short, 10 µl of tear fluid (diluted in 0.9% NaCl) were incubated in the dark with 300 µl Bradford reagent for 10 min and measured with a spectrophotometer at a wavelength of 595 nm. Bovine serum albumin (Thermo Fisher Scientific; 23209) was used to establish a standard curve in each assay.

### ELISA

The IgG concentration in tear fluid samples was analyzed by an enzyme linked immunosorbent assay (ELISA) (abcam, Cambridge, United Kingdom; ab100547). The assay was conducted according to the manufacturer’s protocol, with exception of the standard curve, which was prepared with a range from 10 to 0.08 nanogram per milliliter (ng/ml).

### Analyte concentrations in Schirmer strip samples

Schirmer strip samples were eluted in a known volume of buffer proportional to the collected sample volume to allow conclusions on the original concentration of analytes. Therefore, the wetted distance on the strip was used to calculate the corresponding tear volume with the help of a standard curve. Schirmer strips were incubated with the threefold volume of 0.9% NaCl, thus diluting the sample by factor four. This dilution factor was considered in the subsequent analyses of concentrations.

### Isoelectric focusing and immunoblot

OCB were detected by isoelectric focusing on polyacrylamide gels followed by immunoblotting and detection by IgG-specific antibodies. The procedure was adapted on the basis of the methodology for OCB analysis on polyacrylamide gels with a pH 3–10 gradient reported in^37,38^. Samples were diluted to a concentration of 4 µg/ml, then 10 µl were applied to the gel together with CSF positive and negative controls and focused using a multiphor II electrophoresis system (GE Healthcare, Uppsala, Sweden) for 110 min at 1200 V and 15 milliamperes. Proteins were transferred to a nitrocellulose membrane by passive blotting for 40 min followed by blocking (2% non-fat dry milk/0.9% NaCl) for 30 min. The immuno-detection of IgG was performed using 0.8 µg/ml biotinylated anti-human-IgG-antibody (abcam; ab97223) in 0.2% BSA (Jackson Immunoresearch, Cambridge, United Kingdom; 001-000-161)/0.9% NaCl followed by 0.2 µg/ml HRP-streptavidin (Jackson Immunoresearch; 016-030-84) in 0.4% BSA/0.9% NaCl. All washing steps were performed for ten minutes with 0.9% NaCl. The membrane was incubated in a 3-amino-9-ethylcarbazole substrate solution for 5–15 min until a clear signal of IgG bands was obtained in the positive control sample. The substrate reaction was stopped by removal of the substrate solution and rinsing with water for one minute. Prior to visual inspection and acquisition of digital images membranes were allowed to air dry.

### Equipment and settings

Photographs in Figs. 1 and 2 were taken with a digital reflex camera (Canon EOS 80D; Canon Inc., Ota, Tokyo, Japan) with the setting “close-up”. They were changed to black and white, cut, arranged, and labeled using the program Adobe Photoshop (Version CS4 Windows; Adobe Inc., San José, CA, USA). Figure 4 shows the result of an experiment by isoelectric focusing and immunoblot. The nitrocellulose membrane was scanned with a flatbed scanner (HP Scanjet G3110; HP Inc., Palo Alto, CA, USA) as color image with the resolution 1200 dpi (settings: highlights 20, shadows 0, midtones 0, gamma 1.0) and was saved as TIF-file. The area displayed in Fig. 4 was cut from the full-length image and annotated using the program Adobe Photoshop (Version CS4 Windows; Adobe Inc., San José, CA, USA). No adjustments were made to brightness or contrast. The full-length blot can be found in Supplementary Figure S1.

### Statistical analysis

Statistical analysis and graphical representation of data were performed using GraphPad Prism (version 6.0, GraphPad Software, San Diego, USA; RRID:SCR_002798). Normality of data was assessed by the D'Agostino and Pearson K2 omnibus normality test. The distribution of the majority of data was significantly different from a normal distribution (*p* < 0.05) and therefore non-parametric tests were used. Statistical significance between two paired groups was analyzed by the Wilcoxon matched-pairs signed rank test (two-tailed). Correlation analysis was performed using Spearman rank order correlation to identify linear relationships between two independent parameters. The strength of the association is indicated by Spearman’s rank coefficient *r*_*S*_ (positive or negative) in the following manner: 0.0 ≤ *r*_*S*_ ≤ 0.3 no or very weak correlation; 0.3 < *r*_*S*_ ≤ 0.5 weak correlation; 0.5 < *r*_*S*_ ≤ 0.7 moderate correlation; 0.7 < *r*_*S*_ ≤ 0.9 strong correlation; 0.9 < *r*_*S*_ ≤ 1.0 very strong correlation^39^. The validity of the association for the population was assessed in a significance test, *p* values < 0.05 were considered significant. The 95% confidence intervals (CI) helped to assess the strength of a potential linear relationship^40^.

## Supplementary Information

Supplementary Information.

## Data Availability

All data generated or analyzed during this study are included in this published article and its Supplementary Information files.
