# Supplementary material for: Diagnostic biomarkers in tear fluid: from sampling to preanalytical processing
Source: Sci Rep. 2021 May 12;11:10064. doi: 10.1038/s41598-021-89514-8 (PMC8114924; doi:10.1038/s41598-021-89514-8)
Supplement: Supplementary file 1 — Supplementary Information. [file 41598_2021_89514_MOESM1_ESM.pdf]

Diagnostic biomarkers in tear fluid: from sampling to preanalytical processing

Franziska Bachhuber[1], André Huss[1], Makbule Senel[1], Hayrettin Tumani[1,2*]

[1] Department of Neurology, University Hospital Ulm, Ulm, Germany

[2] Specialty Hospital of Neurology Dietenbronn, Schwendi, Germany

*Corresponding author; hayrettin.tumani@uni-ulm.de

Supplementary Table S1: Flow rate, total protein and IgG concentrations in tear fluid. Tear fluid samples were collected with capillary tubes and Schirmer strips from both eyes consecutively (side I – pause – side II). Time and volume were captured for flow rate determination. IgG concentration was determined by ELISA and total protein concentration by Bradford assay. [a] The volume in μl was calculated from mm on the Schirmer strip scale using a standard curve.

	Capillary tube			Schirmer strip		
**Subject**	**Tear flow rate (μl/min)**	**IgG conc. (μg/ml)**	**Protein conc. (mg/ml)**	**Tear flow rate (μl/min) [a]**	**IgG conc. (μg/ml)**	**Protein conc. (mg/ml)**
1 side I	1.1	83.6	10.6	2.0	107.6	11.9
1 side II	1.5	23.8	13.7	1.1	210.4	15.9
2 side I	2.9	6.5	16.3	4.4	47.8	9.2
2 side II	1.7	2.1	15.1	3.3	32.2	10.1
3 side I	1.1	3.5	14.7	2.9	21.7	9.5
3 side II	1.0	2.5	14.6	1.4	35.9	14.0
4 side I	1.5	206.0	17.9	2.6	99.6	17.3
4 side II	1.4	42.1	16.7	2.1	167.8	19.3
5 side I	1.5	228.0	14.9	1.9	71.8	7.7
5 side II	1.1	134.4	12.4	3.1	85.7	5.8
6 side I	4.1	7.0	16.3	5.7	25.7	6.8
6 side II	4.5	25.6	16.0	2.6	78.7	8.7
7 side I	0.7	49.0	17.6	3.7	36.6	7.6
7 side II	0.9	53.7	11.5	1.7	80.0	10.4
8 side I	2.2	29.6	21.7	3.1	45.8	13.2
8 side II	2.0	21.8	22.3	2.8	75.2	14.9
9 side I	1.0	33.5	14.4	6.3	44.8	4.9
9 side II	0.5	275.0	15.2	5.4	89.9	6.8
10 side I	5.5	8.9	13.4	20.5	13.8	6.2
10 side II	12.8	0.3	10.8	7.0	14.8	5.5

Supplementary Figure S1: Analysis of tear fluid by isoelectric focusing for the detection of oligoclonal IgG bands.

Full-length image of the immunoblot presented in Figure 4.

Tear fluid collected with capillary tubes (**C**) and Schirmer strips (**S**) from the left (**L**) and right (**R**) eye of a healthy subject (subject 4) was analyzed by isoelectric focusing on polyacrylamide gels followed by immunoblot. IgG was detected with biotinylated anti-human-IgG-antibodies followed by HRP-coupled streptavidin and visualized through a substrate reaction with 3-amino-9-ethylcarbazole. CSF samples were used as positive (**+**) and negative (**–**) controls. Tear fluid samples as well as controls were diluted to an IgG concentration of 4μg/ml. Hemoglobin (**Hb**) was used as a crude marker to indicate the position of a pH around 7. Cuts made to select the area displayed in Figure 4 are marked by horizontal dashed black lines.

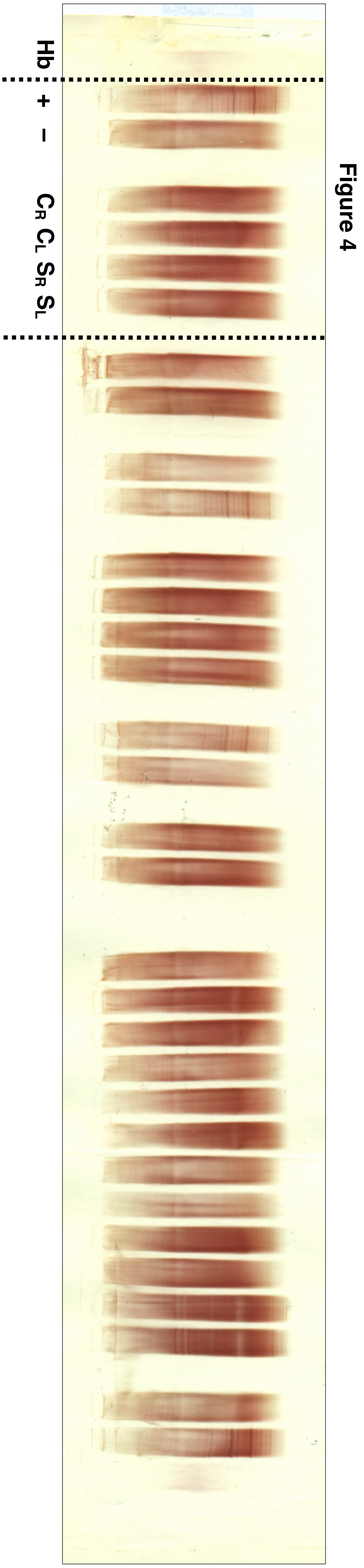

+ positive control (CSF)
– negative control (CSF)

C_R capillary tube – right eye
C_L capillary tube – left eye
S_R Schirmer strip – right eye
S_L Schirmer strip – left eye

Hb hemoglobin
